# Non-*albicans Candida* and rare yeasts in vulvovaginal candidiasis: pathogen attribution, antifungal resistance, persistence, and species-informed management

**DOI:** 10.3389/fmicb.2026.1944046

**Published:** 2026-09-09

**Authors:** Ziyi Yan, Yali Cui, Yingying Li, Xue Xiao, Meng Liao, Xingxin Liu, Wei Zhou, Linghan Kuang, Yongmei Jiang

**Affiliations:** 1 Department of Laboratory Medicine, West China Second University Hospital, Sichuan University, Chengdu, China; 2 Department of Laboratory Medicine, West China Second University Hospital (Tianfu), Sichuan University (Sichuan Provincial Children’s Hospital), Meishan, China; 3 Department of Laboratory Medicine, Meishan Women and Children’s Hospital, Alliance Hospital of West China Second University Hospital, Sichuan University, Meishan, China; 4 Department of Laboratory Medicine, Women and Children’s Hospital of Tibet Autonomous Region, Lhasa, China; 5 Department of Laboratory Medicine, Chengdu Hi-Tech Zone Hospital for Women and Children (Chengdu Hi-Tech Zone Hospital for Maternal and Child Healthcare), Chengdu, China; 6 Key Laboratory of Birth Defects and Related Diseases of Women and Children of Ministry of Education, West China Second University Hospital, Sichuan University, Chengdu, China

**Keywords:** *Candida*, *Candida glabrata*, *Nakaseomyces glabratus*, recurrent vulvovaginal candidiasis, *Saccharomyces cerevisiae*, vulvovaginal candidiasis

## Abstract

Vulvovaginal candidiasis (VVC) is usually attributed to *Candida albicans* (*C. albicans*), but non-*albicans Candida* species and rare yeasts are increasingly encountered in recurrent, complicated, or treatment-refractory disease. Their clinical significance is not uniform: some isolates represent causative infection, whereas others reflect colonization, mixed vaginitis, or detection without causality. This Mini Review addresses three linked clinical and biological questions. First, pathogen attribution should move from detection to causative diagnosis. Recurrent or refractory symptoms warrant structured reassessment during an active episode, integrating clinical examination, vaginal pH and microscopy, fungal culture with species-level identification, exclusion of competing diagnoses, and susceptibility testing when indicated. Second, persistence differs from the *C. albicans* paradigm and may involve niche adaptation, epithelial association, biofilm-associated tolerance, stress survival, and species-dependent relationships between susceptibility phenotypes and persistence-associated traits. Third, management should be species-informed and susceptibility-aware: *Nakaseomyces glabratus* (formerly *Candida glabrata*) and *Candida krusei/Pichia kudriavzevii* require different salvage-treatment logic, whereas evidence for uncommon *Candida* species and rare yeasts remains limited and case-based. New antifungals have promising activity, but current trials do not establish species-specific efficacy in non-*albicans* VVC. Probiotics and microbiome-directed interventions should remain investigational adjuncts evaluated in strain-defined, species-stratified trials rather than substitutes for antifungal therapy. We further identify limitations in the evidence base and priorities for vaginally relevant epithelial, immune-cell, and polymicrobial models. Integrating causative attribution with resistance, tolerance, and persistence biology may reduce both undertreatment of clinically attributable infection and overtreatment of colonization or noninfectious vulvovaginal disease.

## 1 Introduction

Vulvovaginal candidiasis (VVC), typically presenting with vulvar pruritus, soreness, dyspareunia, dysuria, and abnormal vaginal discharge, is one of the most common mucosal fungal infections in women, and recurrent VVC (RVVC) imposes a substantial global burden, affecting an estimated 138 million women annually (Denning et al., 2018). Although *Candida albicans* (*C. albicans*) remains the dominant pathogen, non-*albicans Candida* species represent a clinically important minority, particularly in recurrent, complicated, or treatment-refractory disease. *Candida glabrata* (*C. glabrata*) has been reclassified as *Nakaseomyces glabratus* (Takashima and Sugita, 2022). The clinically familiar name *C. glabrata* is retained throughout this Mini Review because current VVC guidelines and most of the cited clinical studies use that designation. Current U.S. guidelines estimate that *C. glabrata* and other non-*albicans Candida* species are observed in approximately 10%–20% of women with RVVC (Workowski et al., 2021). In a U.S. commercial laboratory dataset, non-*albicans* species accounted for 16% of nearly 10,000 vulvovaginal *Candida* culture results collected during 2019–2023 (Benedict et al., 2024). However, species distributions vary across populations, diagnostic methods, and clinical settings, and the global burden specifically attributable to non-*albicans* VVC remains uncertain. Rare non-*Candida* yeasts are much less frequent and their population-level burden remains poorly defined, but their occurrence in symptomatic vulvovaginal disease further complicates diagnosis and management (Nyirjesy et al., 2022a).

The term “non-*albicans Candida* and rare yeasts” therefore refers to a heterogeneous clinical category rather than a unified pathogenic group. It includes *C. glabrata*, *Candida krusei* (*C. krusei*)/*Pichia kudriavzevii* (*P. kudriavzevii*), *Candida tropicalis* (*C. tropicalis*), the *Candida parapsilosis* (*C. parapsilosis*) complex, *Candida lusitaniae* (*C. lusitaniae*), *Candida kefyr* (*C. kefyr*), and uncommon yeasts such as *Saccharomyces cerevisiae* (*S. cerevisiae*). These organisms differ in morphogenesis, epithelial interaction, biofilm formation, vaginal niche adaptation, and antifungal susceptibility. Their relevance to drug-resistant *Candida* is complementary rather than synonymous with multidrug resistance: intrinsic or acquired susceptibility phenotypes must be interpreted alongside biofilm-associated tolerance, stress adaptation, mucosal persistence, and treatment outcome. This Mini Review therefore asks how causative disease should be distinguished from colonization through structured clinical and microbiological assessment, how species-specific yeasts persist in the vulvovaginal niche, and how organism identity and susceptibility should inform antifungal and adjunctive management. Because the available literature ranges from guidelines and clinical reviews to cohort studies, mechanistic experiments, susceptibility studies, and individual case reports, the strength of inference necessarily differs across organism groups. The species-level evidence and its clinical interpretation are summarized in Table 1.

**TABLE 1:** Species-level evidence for attribution, morphogenesis, resistance/tolerance, persistence, and management in VVC.

Species/group	Clinical relevance and evidence context	Attribution caveats	Resistance, tolerance, and persistence features	Filamentation/morphogenesis	Management implication	References
*Candida glabrata* (*Nakaseomyces glabratus*)	Common clinically important non-*albicans Candida* species in recurrent, complicated, or azole-refractory VVC	May be underdetected by microscopy; culture positivity should be interpreted with symptoms, burden, and repeated recovery	Epithelial adhesion; stress adaptation; reduced azole susceptibility; vaginal biofilm-associated persistence; non-vaginal evidence for host-cell-associated survival; isolate-specific MIC–biofilm correlation	No typical hyphae clinically; condition-dependent pseudohyphal growth *in vitro*	Avoid repeated empirical fluconazole when azole-unresponsive; consider nonfluconazole azoles, boric acid, nystatin, or flucytosine-based salvage	Arastehfar et al., 2023; Chen et al., 2026; Csank and Haynes, 2000; Faria-Gonçalves et al., 2021; Hassan et al., 2021; Pappas et al., 2016; Sobel et al., 2003; Sobel, 2024; Takashima and Sugita, 2022; White et al., 2001; Workowski et al., 2021
*Candida krusei* / *Pichia kudriavzevii*	Less frequent but clinically important because of intrinsic fluconazole resistance	Species-level identification is essential before assuming fluconazole failure is nonspecific recurrence	Intrinsic fluconazole resistance; persistence favored by ineffective empirical exposure	Elongated blastoconidia; branched pseudohyphae	Avoid fluconazole; consider active nonfluconazole topical agents; topical amphotericin B is case-based salvage	Singh et al., 2002; Sobel, 2024; Vempati and Sobel, 2025
*Candida tropicalis*	Non-*albicans Candida* species with epithelial interaction and biofilm-related experimental evidence	Should not automatically be interpreted as *C. glabrata*-like disease	pH-dependent epithelial interaction; biofilm-related phenotypes; cohort-dependent azole susceptibility	Yeast–filament transition; environmentally modulated	Management should consider organism burden, recurrence, MICs, and response to adequate therapy before escalation	Clavijo-Giraldo et al., 2023; Ferreira et al., 2016; Poon and Hui, 2023; Zangl et al., 2020
*Candida parapsilosis* complex	Intermediate clinical significance; reported as pathogen or bystander depending on context	A single low-level culture has limited causal value; reproducibility and response to treatment are important	Adhesion and biofilm formation reported; susceptibility patterns differ from *C. glabrata*	Yeast–pseudohypha transition; environmentally modulated	Use species-level susceptibility and clinical response; avoid automatic escalation to salvage therapy	Borges et al., 2018; Chen et al., 2026; Clavijo-Giraldo et al., 2023; Nyirjesy et al., 2005; Poon and Hui, 2023
Other uncommon *Candida* species, including *Candida lusitaniae* and *Candida kefyr*	Infrequent in VVC; VVC-specific treatment-outcome evidence is limited	Attribution threshold should be higher when species rarity is high or symptoms are discordant	Mechanistic and outcome data are sparse in the vaginal setting	Species dependent; vaginal evidence limited	Management should remain individualized and evidence-limited, relying on repeated recovery, species-level identification, susceptibility testing when indicated, and clinical response	Dennerstein et al., 2011; Farr et al., 2021; Nyirjesy et al., 2022a
*Saccharomyces cerevisiae*	Rare non-*Candida* yeast; evidence mainly from clinical series and case reports	May represent colonization or exogenous introduction; exposure to baking yeast or yeast-containing probiotics should be reviewed	Reduced susceptibility patterns and source-dependent susceptibility have been reported; treatment evidence remains scattered and case-level	Predominantly budding yeast; vaginal filamentation evidence limited	Management should be case-based, using reported clinical cases, exposure history, susceptibility results, and documented clinical–mycological response	Neal et al., 2025; Nyirjesy et al., 1995; Savini et al., 2008; Sobel et al., 1993; Yan et al., 2024

## 2 Pathogen attribution: from detection to causative diagnosis

### 2.1 Compatible syndrome and exclusion of mimics

For non-*albicans Candida* and rare yeasts, pathogen attribution should move from detection to causative diagnosis. Current guidelines diagnose VVC when compatible symptoms and signs are accompanied by microscopic yeasts, hyphae, or pseudohyphae, or by positive culture or another validated yeast test; in complicated or suspected non-*albicans* disease, culture or PCR is recommended to confirm diagnosis and identify the organism (Workowski et al., 2021).

Clinical compatibility is the first attribution step. Vulvar pruritus, soreness, dyspareunia, dysuria, vulvovaginal erythema, and abnormal discharge support a VVC-compatible syndrome, but none is specific. Therefore, evaluation should also address bacterial vaginosis, trichomoniasis, mixed vaginitis, irritant or allergic reactions, inflammatory dermatoses, vulvodynia, and other noninfectious vulvar disorders. This is particularly important for non-*albicans Candida*, because guideline-based management cautions that a substantial proportion of women with non-*albicans Candida*-positive cultures may be minimally symptomatic or asymptomatic, and that other causes of vaginal symptoms should be actively excluded before attributing disease to these organisms (Farr et al., 2021; Saxon et al., 2020; Workowski et al., 2021).

In patients reporting recurrent or refractory VVC, a structured assessment should preferably be performed during an active symptomatic episode and, whenever feasible, before further empirical antifungal treatment is initiated (Neal and Martens, 2022; Rautemaa-Richardson et al., 2026). The clinician should first determine whether previous episodes were evaluated and microbiologically confirmed rather than being defined solely by patient-reported symptoms or self-diagnosis (Neal and Martens, 2022; Rautemaa-Richardson et al., 2026). The timing of each episode, previously administered antifungal agents, dose and duration, treatment adherence, initial clinical response, and interval to symptomatic relapse should also be documented (Farr et al., 2021; Neal and Martens, 2022; Rautemaa-Richardson et al., 2026; Saxon et al., 2020). Vulvar inspection and vaginal examination should then be combined with vaginal pH measurement and saline and/or potassium hydroxide microscopy (Farr et al., 2021; Nyirjesy et al., 2022a; Saxon et al., 2020; Workowski et al., 2021). A vaginal pH of ≤ 4.5 is compatible with VVC, whereas an elevated pH should increase suspicion of bacterial vaginosis, trichomoniasis, or mixed vaginitis, without completely excluding concomitant candidiasis (Nyirjesy et al., 2022a; Saxon et al., 2020; Workowski et al., 2021). Fungal culture with species-level identification should be obtained in recurrent, complicated, atypical, or microscopy-negative disease (Farr et al., 2021; Neal and Martens, 2022; Nyirjesy et al., 2022a; Saxon et al., 2020; Workowski et al., 2021). Targeted testing for bacterial vaginosis, *Trichomonas vaginalis*, and other competing infectious diagnoses should be performed according to the clinical presentation and initial laboratory findings (Farr et al., 2021; Rautemaa-Richardson et al., 2026; Saxon et al., 2020; Workowski et al., 2021). Potential host or treatment-related modifiers—including pregnancy, diabetes mellitus, immunosuppression, recent broad-spectrum antibiotic or corticosteroid exposure, and repeated azole use–should be assessed according to the clinical history rather than through indiscriminate laboratory screening (Akinosoglou et al., 2024a; Farr et al., 2021; Rautemaa-Richardson et al., 2026; Workowski et al., 2021). Finally, antifungal susceptibility testing should be considered when symptomatic, culture-positive disease persists despite an appropriately administered regimen, particularly when a non-*albicans* species or an uncommon yeast is repeatedly recovered (Akinosoglou et al., 2024a; Farr et al., 2021; Nyirjesy et al., 2022a; Saxon et al., 2020; Workowski et al., 2021).

### 2.2 Objective mycological confirmation

Objective mycological confirmation is the pivotal step that separates clinical suspicion from evidence-based attribution. Wet mount microscopy remains useful for rapid diagnosis, but its performance is species dependent. It is most informative when budding yeasts, pseudohyphae, or hyphae are seen in a symptomatic patient, whereas a negative wet mount does not exclude non-*albicans* VVC. *C. glabrata* is the clearest example because typical pseudohyphae or hyphae are not usually observed in routine clinical specimens, making it less readily detected microscopically than *C. albicans* (Workowski et al., 2021). For recurrent, complicated, refractory, or atypical cases, culture should therefore be regarded as more than a confirmatory test.

Vaginal culture on fungal media permits viable recovery, reveals mixed or predominant growth, and allows repeated isolation of the same organism across symptomatic episodes. After growth, isolates can be identified phenotypically or by MALDI-TOF MS, sequencing, or targeted PCR. Unlike direct microscopy or closed molecular panels, culture preserves isolates for susceptibility testing and further characterization, which is valuable when the clinical significance of non-*albicans Candida* or rare yeasts cannot be inferred from detection alone (Farr et al., 2021; Nyirjesy et al., 2022a; Saxon et al., 2020).

Molecular assays add speed and standardization, but their value depends on target coverage. Commercial NAATs and PCR panels can detect common infectious vaginitis syndromes and help exclude bacterial vaginosis or trichomoniasis while identifying common *Candida* targets. Some molecular vaginitis assays report a *Candida* species group, commonly including *C. albicans*, C. *tropicalis*, *C. parapsilosis*, and *Candida dubliniensis* (*C. dubliniensis*), and may separately identify *C. glabrata* and, in some platforms, *C. krusei* (Akinosoglou et al., 2024b; Gaydos et al., 2017; Schwebke et al., 2020; Thompson et al., 2020). In-house or research multiplex PCR assays can distinguish broader Candida panels, including *C. albicans, C. glabrata, C. parapsilosis, C. tropicalis, C. krusei, Candida guilliermondii (C. guilliermondii), C. lusitaniae*, and *C. dubliniensis* (Carvalho et al., 2007). These approaches are valuable when rapid species-level information is needed, but they should not be treated as complete substitutes for culture: targeted panels may miss rare yeasts, and molecular detection alone does not generate an isolate for susceptibility testing, genotyping, biofilm assays, or other persistence-related analyses (Nyirjesy et al., 2022a). Accordingly, a positive molecular result should be interpreted as evidence of organism detection rather than, by itself, proof of causative infection (Akinosoglou et al., 2024b; Neal and Martens, 2022; Nyirjesy et al., 2022a; Rautemaa-Richardson et al., 2026).

### 2.3 Predominance, reproducibility, and species-specific plausibility

After objective confirmation, attribution should be strengthened by evidence of predominance and reproducibility. A single low-level recovery of a non-*albicans* yeast has weaker causal value than moderate-to-heavy growth, predominant recovery in culture, repeated isolation of the same species during symptomatic episodes, or concordance between symptom flares and mycological positivity. Quantitative work has suggested that *Candida*-associated vaginal pathology is more meaningful when fungal burden is considered, rather than interpreting any positive culture as equivalent (Odds et al., 1988). This principle is especially important for rare yeasts or species with uncertain vaginal pathogenicity.

Species-specific plausibility should then refine, rather than replace, objective evidence. *C. glabrata* has an established role in chronic and refractory vaginitis, yet classic clinical data showed that Torulopsis glabrata could be associated with symptomatic vaginitis, asymptomatic carriage, or uncertain clinical significance, and that *in vitro* minimum inhibitory concentrations (MICs) did not always predict azole treatment response (Redondo-Lopez et al., 1990). *C. parapsilosis* represents a more ambiguous intermediate example: it has been described as either a pathogen or a bystander, depending on clinical context, organism persistence, and therapeutic response (Nyirjesy et al., 2005). Broader discussions of non-albicans yeasts similarly caution against assuming pathogenicity from isolation alone (Dennerstein et al., 2011). The appropriate conclusion is therefore not that non-*albicans Candida* and rare yeasts are secondary or clinically unimportant, but that attribution should be proportional to the strength of evidence: the threshold should rise as species rarity increases, organism burden decreases, or symptoms remain discordant with microbiological findings.

In practical terms, causative infection is most strongly supported when a compatible symptomatic episode coincides with objective mycological evidence, alternative infectious and noninfectious diagnoses have been reasonably excluded, and the same organism is recovered predominantly or repeatedly during symptomatic episodes (Farr et al., 2021; Neal and Martens, 2022; Odds et al., 1988; Rautemaa-Richardson et al., 2026; Saxon et al., 2020; Workowski et al., 2021). Conversely, colonization or incidental detection is more likely when the patient is asymptomatic or has an atypical syndrome, the organism is recovered only once at low abundance, another diagnosis better explains the symptoms, or the clinical and mycological findings remain discordant (Dennerstein et al., 2011; Farr et al., 2021; Odds et al., 1988; Rautemaa-Richardson et al., 2026; Saxon et al., 2020; Workowski et al., 2021). These features should be regarded as a clinical attribution framework rather than a validated diagnostic score, because no single finding–including a positive culture, organism burden, or repeated isolation–is sufficient to establish causality in isolation.

### 2.4 Therapeutic response as supportive evidence

Clinical improvement after species-appropriate treatment, particularly when accompanied by mycological clearance, increases confidence that the recovered yeast is clinically relevant. However, persistent symptoms after treatment do not automatically indicate antifungal failure, because recurrent or refractory vulvovaginal symptoms may also reflect relapse after stopping maintenance therapy, resistant or relatively resistant *Candida* species, mixed vaginitis, altered vaginal ecology, inadequate local drug exposure, or noninfectious vulvar disease (Akinosoglou et al., 2024a; Day and Sobel, 2025). Therapeutic response should therefore be regarded as supportive rather than independently diagnostic and should not substitute for baseline mycological confirmation.

From a resistance-focused perspective, a resistant phenotype in a colonizing isolate does not establish resistance-mediated disease, whereas repeated disease caused by a conventionally susceptible isolate may reflect tolerance, biofilm-associated survival, inadequate local exposure, or another diagnosis (Akinosoglou et al., 2024a; Rosenberg et al., 2018; Sherry et al., 2017). Resistance–persistence relationships should therefore be interpreted only after repeated detection has been linked to active disease rather than incidental carriage.

## 3 Persistence mechanisms: shared niche pressures and species-specific strategies

### 3.1 Established mechanisms in *C. albicans* VVC

Most mechanistic studies of VVC have focused on *C. albicans*, in which the transition from commensal carriage to symptomatic mucosal disease involves multiple interacting processes. Adhesion to vaginal epithelial cells, yeast-to-hypha transition, epithelial invasion or damage, candidalysin-associated inflammatory activation, biofilm formation, and host–microbiota interactions have all been implicated in the pathogenesis and recurrence of *C. albicans* VVC (David and Solomon, 2023; Valentine et al., 2025; Willems et al., 2020). These processes occur within a vaginal environment shaped by estrogen status, local innate immunity, lactobacilli, acidic pH, organic acids, and nutrient availability.

The persistence of non-*albicans Candida* and rare yeasts cannot be explained by simply extending this *C. albicans*-centered framework. Several clinically relevant non-*albicans* species lack prominent hyphal morphogenesis, differ in epithelial interaction or inflammatory activation, or are encountered mainly in azole-refractory or recurrent disease. Persistence is therefore better interpreted through species-dependent combinations of niche adaptation, epithelial association, biofilm-associated tolerance, stress survival, and antifungal resistance.

### 3.2 Niche adaptation and epithelial persistence

For non-*albicans Candida*, persistence begins with the capacity to remain physiologically fit in the vulvovaginal niche and to maintain epithelial association under local stress. Clinically relevant species, including *C. glabrata*, *C. parapsilosis*, *C. krusei*, and *C. tropicalis*, respond differently to lactic acid stress, indicating that the acidic, organic-acid-rich vaginal environment may impose species-specific selective pressures (Zangl et al., 2020). Low-pH organic-acid conditions can also influence the growth and azole response of *C. glabrata*, suggesting that apparent persistence after treatment may partly reflect niche-dependent physiological adaptation rather than resistance alone (Lourenço et al., 2019).

Epithelial persistence is likewise species dependent. *C. glabrata* provides a clear contrast to hypha-dominant pathogenicity: typical hyphae are not observed in routine clinical specimens, although pseudohyphal growth can be induced experimentally under nitrogen-starvation conditions (Csank and Haynes, 2000). The relevance of this experimental transition to vaginal disease remains uncertain; clinically relevant persistence is more plausibly linked to adhesin-mediated host-cell association, stress adaptation, immune evasion, and reduced azole susceptibility (Hassan et al., 2021). Intracellular survival and a macrophage-induced, drug-tolerant persister state have also been demonstrated in non-vaginal macrophage models (Arastehfar et al., 2023). *C. tropicalis* can interact with human epithelium in a pH-dependent manner, suggesting that local environmental conditions influence its epithelial association and invasion potential (Ferreira et al., 2016). These findings support a more specific interpretation of niche adaptation in non-*albicans* VVC: persistence is not simply continued growth in the vagina, but the ability of a given species to remain viable, epithelial-associated, and clinically relevant within a locally permissive or disturbed mucosal environment.

### 3.3 Biofilm-associated persistence: evidence and limits

Biofilm formation is one of the most frequently proposed mechanisms linking recurrence, persistence, and reduced antifungal response in VVC. In the non-*albicans* context, this mechanism is particularly relevant because biofilm-associated cells may display reduced susceptibility despite the absence of classical planktonic resistance. RVVC has been proposed as an interkingdom process involving *Candida* and *Lactobacillus* communities (McKloud et al., 2021). Cell-free products from selected lactobacilli can inhibit biofilm formation or filamentation in *C. albicans*, *C. tropicalis*, and *C. parapsilosis*, although the effects vary by bacterial strain and fungal species (Poon and Hui, 2023). Conversely, *Lactobacillus iners* cell-free supernatant enhanced biofilm formation and hyphal/pseudohyphal growth in selected *C. albicans* vaginal isolates (Sabbatini et al., 2021). These contrasting *in vitro* findings suggest that bacterial effects depend on community composition, strain identity, fungal species, and secreted metabolite profiles rather than on *Lactobacillus* abundance alone. They support community-dependent ecological modulation but do not establish stable fungal–bacterial co-adaptation; direct evidence that dysbiosis-associated metabolites promote non-*albicans Candida* persistence *in vivo* remains limited.

Clinical and experimental studies provide supportive, but not uniform, evidence for this mechanism. Isolates from RVVC patients can form heterogeneous biofilms with reduced fluconazole susceptibility (Sherry et al., 2017), and recent work has reported morphologically distinct *Candida* biofilms on human vaginal epithelium in RVVC (Pan et al., 2023). A prospective clinical study further supports a role for *Candida* biofilm features in RVVC pathogenesis, although relationships among biofilm formation, symptoms, recurrence, and treatment outcome remain incompletely resolved (Díaz-Navarro et al., 2025). For individual non-*albicans* species, *C. parapsilosis* has been studied for adhesion and biofilm formation on abiotic surfaces, including copper intrauterine device fragments (Borges et al., 2018), and N-linked mannosylation contributes to biofilm formation in both *C. parapsilosis* and *C. tropicalis* (Clavijo-Giraldo et al., 2023).

At the same time, biofilm should not be presented as a universal explanation for recurrent or refractory VVC. Histological analysis of VVC lesions has suggested that some lesions are primarily invasive and polymicrobial rather than classical *Candida*-containing biofilms (Swidsinski et al., 2019). Therefore, biofilm-associated persistence should be interpreted conditionally: it may be important in selected patients, species, or microenvironments, but it should be evaluated alongside attribution strength, organism predominance, recurrence pattern, species identity, and antifungal response.

### 3.4 Antifungal resistance, tolerance, and virulence under treatment pressure

Antifungal resistance and tolerance are related but distinct contributors to persistence: resistance is reflected by reduced susceptibility on conventional testing, whereas tolerance denotes survival or slow growth during drug exposure without necessarily shifting the MIC (Rosenberg et al., 2018). Thus, repeated recovery after azole exposure may reflect biofilm-associated tolerance, stress adaptation, residual organisms, inadequate local exposure, or true resistance (Akinosoglou et al., 2024a; Sherry et al., 2017). A recent secondary analysis of a multicenter trial found that baseline vaginal-microbiota categories were associated with differences in short-term clinical efficacy and also examined 30-day mycological recurrence after topical azole treatment (Mi and Zhang, 2025). However, these associative data neither demonstrate direct microbial metabolism or inactivation of antifungal agents nor establish a non-*albicans*-specific effect. A vaginal pharmacomicrobiome contribution should therefore be considered a testable hypothesis rather than an established explanation for recurrent non-*albicans* VVC. *C. krusei/P. kudriavzevii* illustrates intrinsic fluconazole resistance, whereas *C. glabrata* spans reduced azole susceptibility, acquired resistance, and stress tolerance; persister-like states have been demonstrated in non-vaginal macrophage models and should not be assumed to operate identically in VVC (Arastehfar et al., 2023; Hassan et al., 2021; Singh et al., 2002; Vempati and Sobel, 2025).

Recent vaginal surveillance further demonstrates that antifungal resistance among non-*albicans Candida* is strongly species and drug dependent. In a 2019–2024 Shanghai cohort comprising 7,894 clinically and laboratory-confirmed VVC episodes, the reported resistant or non-wild-type proportions–depending on the available interpretive criteria—among 1,392 vaginal *C. glabrata* isolates were 1.87% for fluconazole, 5.32% for itraconazole, and 24.28% for voriconazole (Chen et al., 2026). The corresponding proportions in *C. tropicalis* were 33.70%, 22.83%, and 27.17%, respectively, whereas fluconazole and voriconazole resistance in *C. parapsilosis* was detected in 2 of 121 isolates (1.65%) for each agent (Chen et al., 2026). These estimates should be regarded as cohort- and method-specific rather than as universal resistance rates for non-*albicans Candida*.

The relationship between drug-response phenotypes and virulence is context dependent rather than uniformly positive. In sequential vaginal isolates from chronic cases, increasing MICs and biofilm formation were correlated only in *C. glabrata* (Faria-Gonçalves et al., 2021). This observation supports a possible link between evolving drug-response phenotypes and persistence-associated traits in selected *C. glabrata* populations, but it does not show that resistance generally increases tissue invasion, inflammation, or clinical severity across non-*albicans Candida*. Vaginal, species-resolved evidence is therefore required before resistance–virulence relationships observed in systemic or non-vaginal models are extrapolated to VVC.

### 3.5 Rare yeasts: exogenous introduction versus endogenous persistence

Rare non-*Candida* yeasts create a different persistence problem: repeated detection may reflect endogenous mucosal persistence, intermittent colonization, or repeated exogenous introduction. *S. cerevisiae* has been recovered in both asymptomatic carriage and symptomatic vaginitis, with source-dependent susceptibility patterns (Sobel et al., 1993; Yan et al., 2024). Baking-yeast transmission and a probiotic-associated recurrent case indicate that exposure to baking yeast or yeast-containing probiotic products can be mechanistically relevant (Neal et al., 2025; Nyirjesy et al., 1995). Other organism-specific treatment evidence remains case-based (Savini et al., 2008). Recurrent recovery should therefore prompt review of baking or occupational contact and yeast-containing supplements before it is attributed to an intrinsic persistence phenotype.

## 4 Susceptibility-guided management: evidence-based decisions and unresolved controversies

### 4.1 From attribution to actionable treatment decisions

The management of non-*albicans Candida* and rare yeasts should begin after a causative role has been reasonably established. At this stage, targeted therapy should be justified by concordance among species identity, susceptibility profile, organism persistence, prior antifungal exposure, and clinical course. Susceptibility-guided management is therefore not simple MIC-based drug selection, but a decision process that links attribution, persistence biology, and the limited antifungal options available for non-*albicans Candida* vaginitis (Akinosoglou et al., 2024a; Sobel, 2024). Accordingly, the following section separates guideline-supported approaches for common non-*albicans Candida* species from case-based management considerations for rare yeasts, for which organism-specific treatment algorithms remain unavailable.

### 4.2 Species-informed antifungal strategies

For symptomatic non-*albicans* VVC after competing diagnoses have been excluded, current guidance generally favors a longer course of a nonfluconazole azole, usually for 7–14 days, rather than repeated short-course fluconazole. If recurrence occurs, intravaginal boric acid, commonly 600 mg daily for 3 weeks, is recommended in CDC guidance and has reported clinical and mycological eradication rates of approximately 70%, although the optimal treatment of non-*albicans* VVC remains uncertain (Workowski et al., 2021). This recommendation should be interpreted as a pragmatic strategy rather than a universal solution: treatment success varies by species, fungal burden, host factors, prior azole exposure, and whether the recovered organism is truly causative (Akinosoglou et al., 2024a; Donders et al., 2022; Nyirjesy et al., 2022a; Satora et al., 2023; Sobel, 2024).

*C. glabrata* requires particular caution because reduced azole susceptibility and the lack of typical hyphae in routine clinical specimens complicate both treatment and microscopic detection. For azole-unresponsive, clinically attributable infection, IDSA-listed options include intravaginal boric acid 600 mg daily for 14 days, nystatin suppositories 100,000 units daily for 14 days, or compounded 17% flucytosine cream alone or with 3% amphotericin B cream daily for 14 days; CDC guidance instead uses a 3-week boric acid course for recurrent non-*albicans* VVC (Pappas et al., 2016; Sobel, 2024; Workowski et al., 2021). In a retrospective series of symptomatic *C. glabrata* vaginitis, boric acid achieved clinical and mycological success in 47/73 (64%) and 27/38 (71%) women at two centers, whereas nightly flucytosine for 14 days succeeded in 27/30 (90%) women after azole and boric acid failure (Sobel et al., 2003). For highly refractory disease, compounded intravaginal flucytosine 1 g plus amphotericin B 100 mg once daily for 14 days produced clinical and microbiological improvement in three reported cases (White et al., 2001). These regimens require individualized specialist supervision and should not be used for colonization or discordant culture positivity.

*C. krusei*/*P. kudriavzevii* represents a different therapeutic problem. Because intrinsic fluconazole resistance is central to its clinical behavior, repeated empirical fluconazole is biologically implausible once this species is identified. Management should instead rely on nonfluconazole topical azoles or other local regimens guided by susceptibility, clinical response, and specialist judgment, while recognizing that high-quality trial data remain sparse (Singh et al., 2002; Sobel, 2024; Vempati and Sobel, 2025). In refractory *C. krusei* vaginitis, topical amphotericin B has been reported as a successful case-based option (Chamorro-de-Vega et al., 2016), but this should be presented as salvage evidence rather than as a standard regimen. This distinction is clinically important: salvage therapy for *C. krusei/P. kudriavzevii* primarily requires avoidance of fluconazole and selection of an active topical agent, whereas *C. glabrata* more often requires boric acid, nystatin, or compounded flucytosine-based therapy (Pappas et al., 2016; Singh et al., 2002; Sobel, 2024; Vempati and Sobel, 2025).

By contrast, *C. tropicalis* and the *C. parapsilosis* complex should not automatically be managed as *C. glabrata*-like disease. Antifungal susceptibility data from vaginal yeast isolates show species-dependent susceptibility patterns, supporting species-level interpretation rather than uniform escalation for all non-*albicans Candida* isolates (Chen et al., 2026). These species may remain susceptible to standard azoles, but recurrence, high organism burden, biofilm-associated phenotypes, elevated MICs, or failure of adequate therapy should prompt individualized interpretation rather than automatic escalation.

For *S. cerevisiae*, no species-specific treatment algorithm exists. Treatment should follow repeated or predominant recovery in a compatible syndrome, review of exogenous sources, susceptibility testing when feasible, and documentation of clinical–mycological response (Savini et al., 2008; Sobel et al., 1993; Yan et al., 2024). Oral fluconazole succeeded in two itraconazole-resistant cases (Savini et al., 2008), and intravaginal boric acid in one probiotic-associated recurrent case (Neal et al., 2025), but these reports do not establish a general regimen. Rare yeasts should therefore remain case-based comparators rather than extensions of *Candida*-specific resistance mechanisms or drug-development pathways.

### 4.3 New antifungals and translational priorities for resistant *Candida*

Oteseconazole and ibrexafungerp illustrate complementary development strategies: increased fungal CYP51 selectivity and an oral non-azole glucan-synthase mechanism, respectively. In the two VIOLET trials, following fluconazole induction, oral oteseconazole 150 mg daily for 7 days and then once weekly for 11 weeks reduced the proportions of participants experiencing recurrence through week 48 to 6.7% and 3.9%, compared with 42.8% and 39.4% in the corresponding placebo groups (Sobel et al., 2022). Acute VVC efficacy of ibrexafungerp has been evaluated using 300 mg orally twice in 1 day (Nyirjesy et al., 2022b; Schwebke et al., 2022); in VANISH 303, clinical cure and mycological eradication were achieved in 50.5% and 49.5% of participants, compared with 28.6% and 19.4% receiving placebo, respectively (Schwebke et al., 2022). In the CANDLE trial, the same 1-day ibrexafungerp regimen repeated every 4 weeks for six treatments resulted in no mycologically proven recurrence in 70.8% versus 58.5% with placebo and clinical success in 65.4% versus 53.1% at week 24 (Goje et al., 2025). Ibrexafungerp also retained *in vitro* activity at pH 4.5 against fluconazole-susceptible and -resistant vaginal isolates spanning *C. albicans, C. glabrata, C. krusei, C. parapsilosis*, and *C. tropicalis* (Sobel et al., 2021). However, these trials predominantly enrolled *C. albicans* VVC and do not establish species-specific clinical efficacy in non-*albicans* VVC or activity against rare non-*Candida* yeasts. Future trials should therefore report species-stratified clinical cure, mycological eradication, and recurrence rather than extrapolating efficacy from predominantly *C. albicans* populations.

### 4.4 Microbiome-directed adjuncts and exposure confounding

Microbiome-directed approaches should be separated from antifungal treatment. Although *Lactobacillus*-based products may plausibly modify vaginal pH, epithelial adhesion, or biofilm formation, CDC guidance finds insufficient evidence for their use as treatment for VVC, and recent clinical studies remain heterogeneous in probiotic strains, formulations, dosing schedules, patient populations, and outcome definitions; non-*albicans Candida* remains underrepresented (Abavisani et al., 2024; Akinosoglou et al., 2024c; Workowski et al., 2021). Candidate strains for future trials should first undergo strain-level characterization and reproducible screening against clinically relevant non-*albicans* isolates under acidic, vaginally relevant conditions, including epithelial adhesion and biofilm assays (Akinosoglou et al., 2024c; Poon and Hui, 2023). Subsequent randomized trials should enroll patients with clinically and microbiologically confirmed VVC or RVVC and compare standard antifungal therapy plus a defined probiotic with standard antifungal therapy plus placebo, rather than evaluating probiotics as substitutes for antifungal treatment (Abavisani et al., 2024; Akinosoglou et al., 2024c). Randomization should be stratified by *Candida* species and recurrence status, with prespecified outcomes including clinical cure, mycological eradication, time to recurrence, adverse events, and longitudinal changes in the vaginal microbiome and probiotic-strain persistence. Yeast-containing products require additional caution: repeated *S. cerevisiae* recovery should prompt review and discontinuation of a plausible product source, as illustrated by a case report (Neal et al., 2025). Probiotics should therefore remain investigational adjuncts and should not delay culture, species identification, or indicated antifungal therapy.

## 5 Evidence gaps and future directions

### 5.1 Clinical interpretation of resistance and persistence

Non-*albicans Candida* VVC is a mucosal setting in which pathogen attribution, antifungal response, and persistence biology must be interpreted together. A resistant isolate may be clinically irrelevant when it represents colonization, whereas a conventionally susceptible isolate may persist through biofilm-associated tolerance, stress adaptation, residual organisms, or inadequate local exposure. Available vaginal data suggest that reduced susceptibility can coincide with persistence-associated traits in selected *C. glabrata* populations (Faria-Gonçalves et al., 2021), but neither non-*albicans* identity, recurrence, nor fluconazole failure is equivalent to multidrug resistance. The direction and clinical consequences of resistance–virulence coupling remain species-, mechanism-, and niche-dependent. Longitudinal, species-resolved cohorts with paired vaginal isolates obtained during symptomatic episodes, after treatment, and at recurrence are therefore needed to distinguish stable colonization, microbiological persistence, reinfection, and within-host evolution. Such cohorts should integrate species identification, standardized susceptibility testing, tolerance and biofilm phenotyping, antifungal exposure, symptom status, and mycological outcomes to determine when resistance-associated changes are actionable drivers of persistence. Antifungal-development programs should include clinically attributable non-*albicans* infections and report species-stratified clinical cure, mycological eradication, and recurrence rather than extrapolating from predominantly *C. albicans* populations. Rare non-*Candida* yeasts should remain boundary comparators: they reveal the limits of *Candida*-centered diagnostics and therapy but do not justify extending *Candida* resistance mechanisms or treatment evidence beyond the genus.

### 5.2 Optimizing vaginally relevant *in vitro* models

Conventional planktonic assays are valuable for defining individual mechanisms but incompletely reproduce the vaginal niche. Susceptibility and tolerance assays should therefore incorporate acidic pH, lactic and acetic acids, physiologically relevant nutrient conditions, and, where appropriate, microaerophilic conditions, because *Candida* species respond differently to these environmental stresses and their effects on azole activity are species-dependent (Lourenço et al., 2019; Zangl et al., 2020). Epithelial systems should progress from monolayers to three-dimensional or organotypic vaginal models and measure adhesion, epithelial damage, cytokine production, and time-resolved host–fungal transcriptional responses. Multispecies epithelial experiments have demonstrated species- and time-dependent responses to *C. albicans, C. glabrata, C. parapsilosis*, and *C. tropicalis* (Pekmezovic et al., 2021), while experiments using clinical isolates have identified divergent EGFR/MAPK-mediated inflammatory responses to *C. albicans* and non-*albicans Candida* species (Zhang et al., 2022). For questions concerning vaginal immunity, epithelial models should be complemented with defined immune-cell components, particularly neutrophils, rather than assuming that epithelial readouts alone represent the mucosal immune response. Dominant *Lactobacillus* species and, when clinically relevant, dysbiosis-associated bacteria should also be incorporated because interkingdom interactions and bacterial secreted products can alter *Candida* biofilm behavior and filamentation (McKloud et al., 2021; Poon and Hui, 2023; Sabbatini et al., 2021). Such polymicrobial systems should use defined bacterial consortia or cell-free metabolite mixtures to test their effects on non-*albicans* yeast growth, morphogenesis, biofilm formation, and local antifungal activity. To improve reproducibility and translational relevance, studies should use multiple well-characterized clinical isolates per species, include paired pretreatment and post-treatment isolates, model repeated antifungal exposure and biofilm growth, and distinguish resistance from tolerance. Experimental findings should ultimately be validated against patient-level measures of symptoms, fungal burden, inflammatory markers, mycological clearance, and recurrence.

### 5.3 Limitations of the evidence base and this mini review

This Mini Review has several limitations. First, the available evidence is unevenly distributed: *C. glabrata* accounts for much of the clinical and mechanistic literature on non-*albicans* VVC, whereas uncommon *Candida* species and non-*Candida* yeasts are represented predominantly by small observational studies, case series, or individual reports. Second, differences in case definitions, sampling time points, species-identification methods, susceptibility-testing platforms, and interpretive criteria limit direct comparison of prevalence, resistance, and treatment outcomes across studies. Third, many mechanistic findings derive from single strains, non-vaginal experimental conditions, or *C. albicans*-centered systems and may not be directly transferable to symptomatic non-*albicans* VVC. The proposed framework should therefore be interpreted as an evidence-informed clinical and research synthesis rather than a validated diagnostic or therapeutic algorithm.

## 6 Conclusion

Non-*albicans Candida* and rare yeasts should not be managed as a uniform extension of *C. albicans* VVC. Detection becomes clinically actionable only when a compatible syndrome, exclusion of competing diagnoses, mycological confirmation, and organism predominance or reproducibility support causative attribution. In recurrent or refractory disease, species identification and, when indicated, susceptibility testing should be interpreted alongside antifungal exposure, clinical response, and persistence biology, because resistance, tolerance, biofilm-associated survival, and niche adaptation are overlapping but non-equivalent phenomena. Management should therefore be species-informed and susceptibility-aware: repeated empirical fluconazole should give way to appropriately selected regimens, while rare yeasts generally require case-based decisions. This integrated approach can reduce both undertreatment of clinically attributable infection and overtreatment of colonization, incidental detection, or noninfectious vulvovaginal disease.
